# Abdominoplasty: An Easy Approach to Giant Abdominal Lipomas

**DOI:** 10.1155/2020/7875169

**Published:** 2020-02-11

**Authors:** Bayan Alsharif, Hatan Mortada, Aeshah Mandili, Fahad Aljindan

**Affiliations:** ^1^College of Medicine, Umm Alqura University, Makkah, Saudi Arabia; ^2^Department of Plastic Surgery & Burn Unit, King Saud Medical City, Riyadh, Saudi Arabia; ^3^King Abdullah Medical City, Makkah, Saudi Arabia

## Abstract

*Introduction*. Giant lipomas, which are greater than 10 cm, are rare, cosmetically unacceptable, and deteriorate the quality of daily living. Removal of giant abdominal lipomas either by liposuction, excision, or both, can lead to the formation of a loose, pendulous drooping abdomen, and abdominal wall laxity, which is aesthetically displeasing. The objective of this case report is to highlight an easy approach to treat giant abdominal lipoma through therapeutic abdominoplasty. *Case History*. In this case, a 29-year-old man with a known case of hypothyroidism and HCV was in remission but had a huge abdominal mass on his lower left side; it progressed for 7 years and increased in size and caused discomfort. His BMI was 29.53 and the mass measured about 15 × 13 cm. All other investigations were normal and showed no malignancies. He underwent excision of the giant abdominal lipoma using a standard abdominoplasty approach. *Conclusion*. In conclusion, in selected patients, giant abdominal lipomas can be successfully excised along with the redundant abdominal skin.

## 1. Introduction

Lipomas are common benign mesenchymal tumors made of lobulated, mature adipose tissue, which typically lie in the subcutaneous tissues and are commonly encapsulated by a thin, fibrous capsule detached from the underlying muscle fascia. Lipomas are generally soft, movable, painless, slow-growing masses. They are often small, with a diameter less than 5 cm and may only be removed by simple excision if they are painful, uncomfortable, or restrict movement due to their location on joints or for cosmetic reasons [1]. Lipomas can sometimes be “large,” defined as equal to or greater than 5 cm in diameter and can rarely be “giant” when sized at least 10 cm or weigh a minimum of 1000 g [2]. Giant lipomas in the head, neck, back, abdomen, and extremities cause cosmetically unacceptable gibbosity, problems in daily living, and deteriorate quality of life. Mass localization also can restrict body functions and movements [3].However, giant lipomas have a relatively high malignant potential; therefore, surgical excision is the treatment of choice to alleviate the symptoms and apprehend malignancy and provide tissue diagnosis [4]. Liposuction, combined liposuction and excision, has been used in multiple reports with successful lipoma removal [5]. Removal of bulging abdominal giant lipomas either by liposuction, excision, or both, can lead to the formation of a loose, pendulous drooping abdomen, and abdominal wall laxity, which is aesthetically unpleasing. To the best of our knowledge, there have been no previous reports of an abdominoplasty for a patient presenting a giant abdominal lipoma. As such, it is crucial to perform the best suitable operation for each case to achieve the best function and aesthetic results for the patient. This report presents a case of a young man who suffered from a huge abdominal mass and underwent abdominoplasty, resulting in remarkable functional and cosmetic improvement in his health and quality of life.

## 2. Case Presentation

### 2.1. History

A 29-year-old man with a known case of hypothyroidism and HCV was in remission. He was referred to the hospital because of a huge abdominal mass on his lower left side that had been there for 7 years. It was associated with discomfort and heaviness, progressively increased in size, yielded no pain, no other swelling, no history of trauma or surgeries, and no constitutional symptoms. Therefore, otherwise, the patient was healthy.

### 2.2. Physical Examination

**Figure 1:**
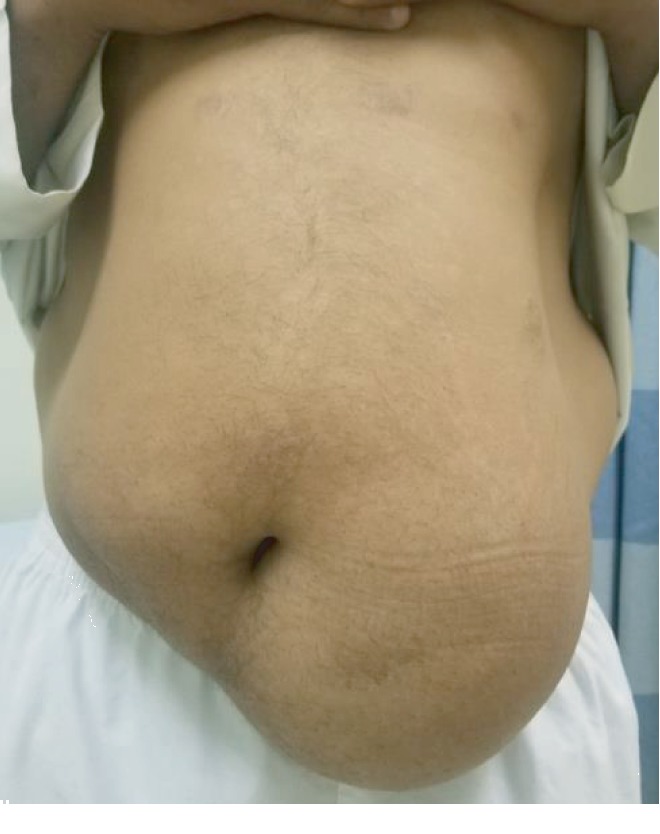
The patient (frontal view) before surgery.

The patient's BMI was 29.53. The mass measured about 15 × 13 cm in the left lower abdomen (Figure 1). There were no signs of infection or skin abnormalities; the mass was soft and mobile, tethered to the skin, and was not pulsatile or tender. There were no abdominal hernias.

### 2.3. Investigation

Blood work was within normal limits. An MRI abdomen with contrast was taken and showed subcutaneous fat tissue with no worrisome underlying mass or abdominal enhancement to suggest liposarcoma. There was no definitive evidence of an abdominal wall hernia. The visualized portion of the pelvic organs and bones appeared to be grossly normal. The patient was admitted to the surgical ward for surgical excision of the mass and provided consent for the surgery.

### 2.4. Surgical Technique

**Figure 2:**
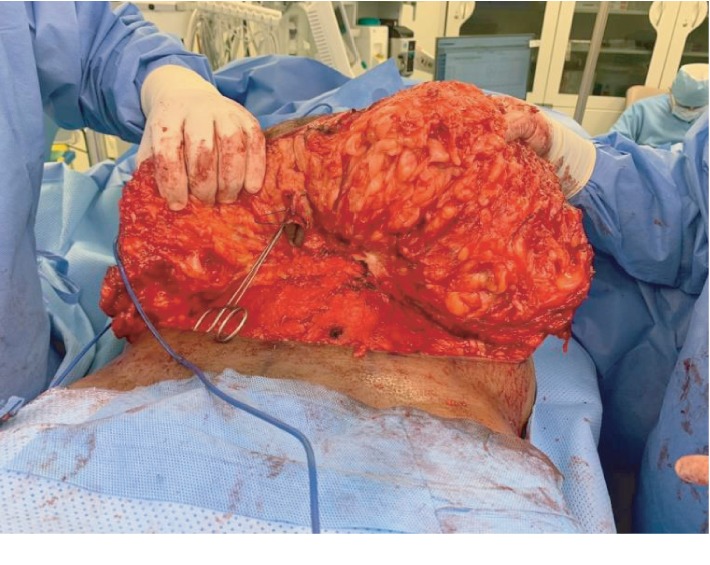
Intraoperative. Raised flap.

**Figure 3:**
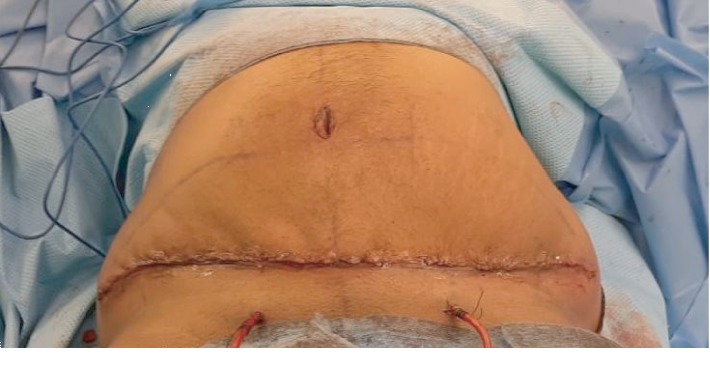
After skin closure.

**Figure 4:**
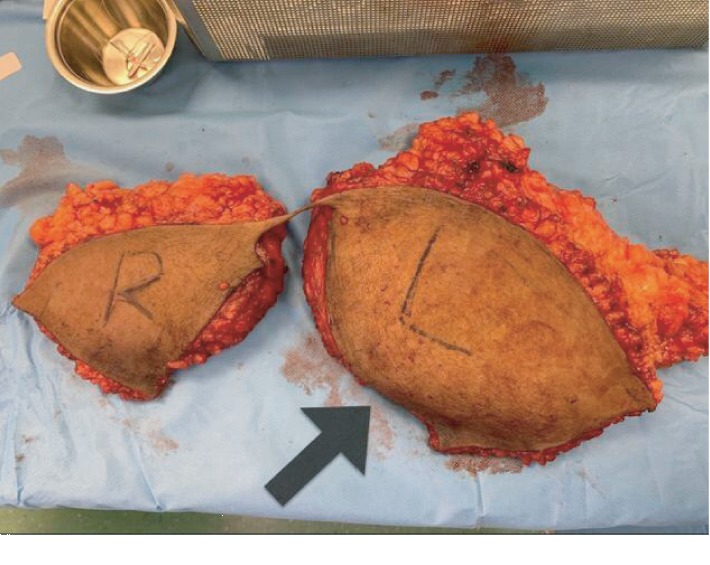
Excised specimen. Weight, 2100 g.

The procedure was carried out under general anesthesia. The patient was placed in a supine position on the operating table, and the abdomen was prepared and draped in the standard sterile manner. A lower abdominal incision 8 cm from the root of the penis extended to the level of the anterior superior iliac spine bilaterally was made. The abdominal flap was undermined to a level just above the rectus fascia. Once the umbilicus was reached, it was freed from the raised abdominal flap circumstantially and the flap was raised to the level of the costal margin (Figure 2). The mass was found to be diffused and arising from the subcutaneous tissue with no definite capsule. The mass was included and completely resected along with the excess abdominal skin. Hemostasis was secured, as two suction drains were inserted and brought out through the pubic area. Finally, the skin was approximated and closed in three layers using 0 vicryl for the Scarpas fascia, 2.0 vicryl for the deep dermal layer, and 3.0 monocryl for the subcuticular layer (Figure 3). The wound was covered with a single layer of Dermabond and an abdominal binder was applied. The weight of the excised mass was about 2 kilograms (Figure 4).

### 2.5. Histopathology Analysis

The specimen showed lipoma in the left side of the abdomen, which consisted of an oriented fibrofatty tissue measuring 20 × 20 × 10 cm and elliptical skin measuring 22 × 14 cm.

### 2.6. Hospital Course

The patient had an uneventful hospital course and was discharged on the first postoperative day in a stable condition.

### 2.7. Follow-Up

**Figure 5:**
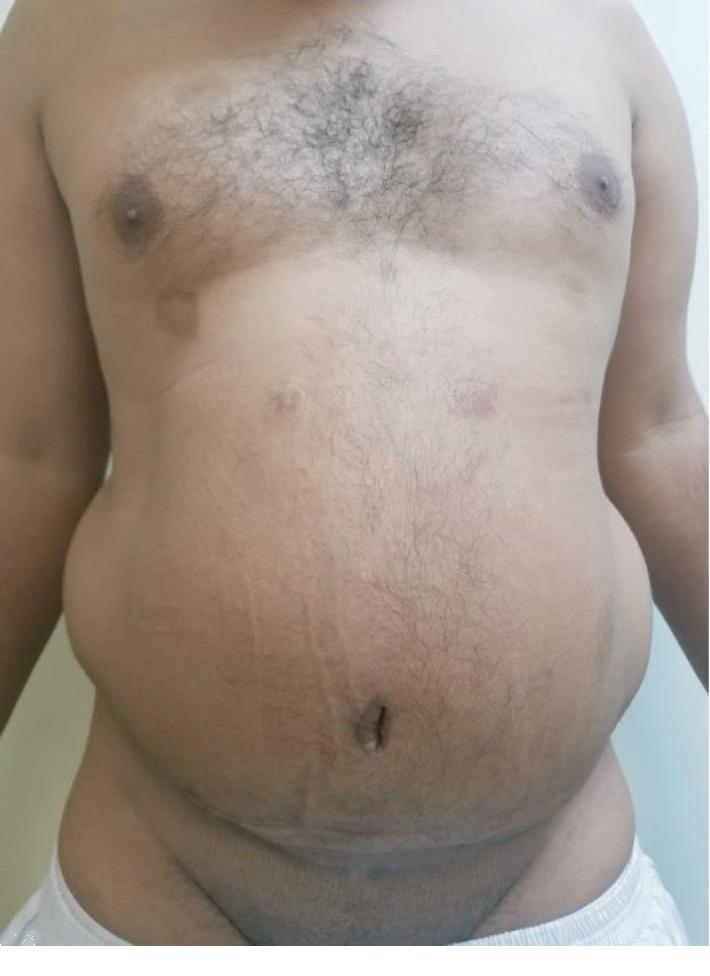
2 months postoperative result.

The patient was seen in the clinic 2 days after discharge, 7 days later, and 2 months after surgery (Figure 5). The drains were removed once the drainage output was less than 30 mL in 24 hours. No complications were encountered.

## 3. Discussion

A single lipoma is well known to be the most common cause of soft tissue tumors, which usually appears between the age of 40 and 60 years old [6]. A “giant lipoma” is considered when the diameter is at least 10 cm or weighs a minimum of 1 kilogram [6, 7], as in the patient in our study. The weight and diameter of the giant lipoma were the main cause of the patient's poor quality of life. Diagnosis of such a mass is mainly clinical. However, malignancy, which can be a significant problem, must be ruled out first. In addition to FNAC, MRI is a well-established tool when it comes to surgical planning and diagnosis [8]. In our case, the benign nature of the lipoma was confirmed by histopathology and MRI. According to the literature, any soft tissue mass with a diameter more than 5 cm is considered malignant until proven otherwise [9]. In this patient, the lipoma was about 15 × 13 cm, but luckily histopathology showed a benign mass. The treatment of choice for these large tumors is always surgical excision [8]. Many methods have been established to manage giant lipomas, for example, direct removal, removal through a small incision [10], endoscopic removal [11], liposuction [12, 13], and laser extirpation [12, 14]. Many other articles have stated that liposuction can actually improve the early aesthetic results, reduce the risk of postoperative hematoma and seroma formation, and reduce operative time [12, 13, 15]. Liposuction was not used in this case report, because the recurrence risk was higher when compared to conventional removal [15]. In addition, there is a possibility of malignant transformation and the concern that excision may be incomplete. Copeland-Halperin et al. concluded that removal by combined liposuction and direct excision is a reasonable alternative to direct, open excision [5]. In the available literature, undergoing abdominoplasty is a common procedure for cosmetic reasons and is used worldwide. However, there are very few cases that have been reported for choosing abdominoplasty for therapeutic advantages in addition to being cosmetic. Taylor et al. have recently published a prospective multicenter study that states a significant improvement in low back pain and urinary incontinence following abdominoplasty among 214 patients. In this case report, we describe a patient who underwent excision of a giant abdominal lipoma using a standard abdominoplasty approach, which resulted in significant improvement in the quality of life and relieved most of his complains.

## 4. Conclusion

In conclusion, in selected patients, giant abdominal lipomas can be successfully excised along with the surrounding stretched and redundant abdominal skin.
